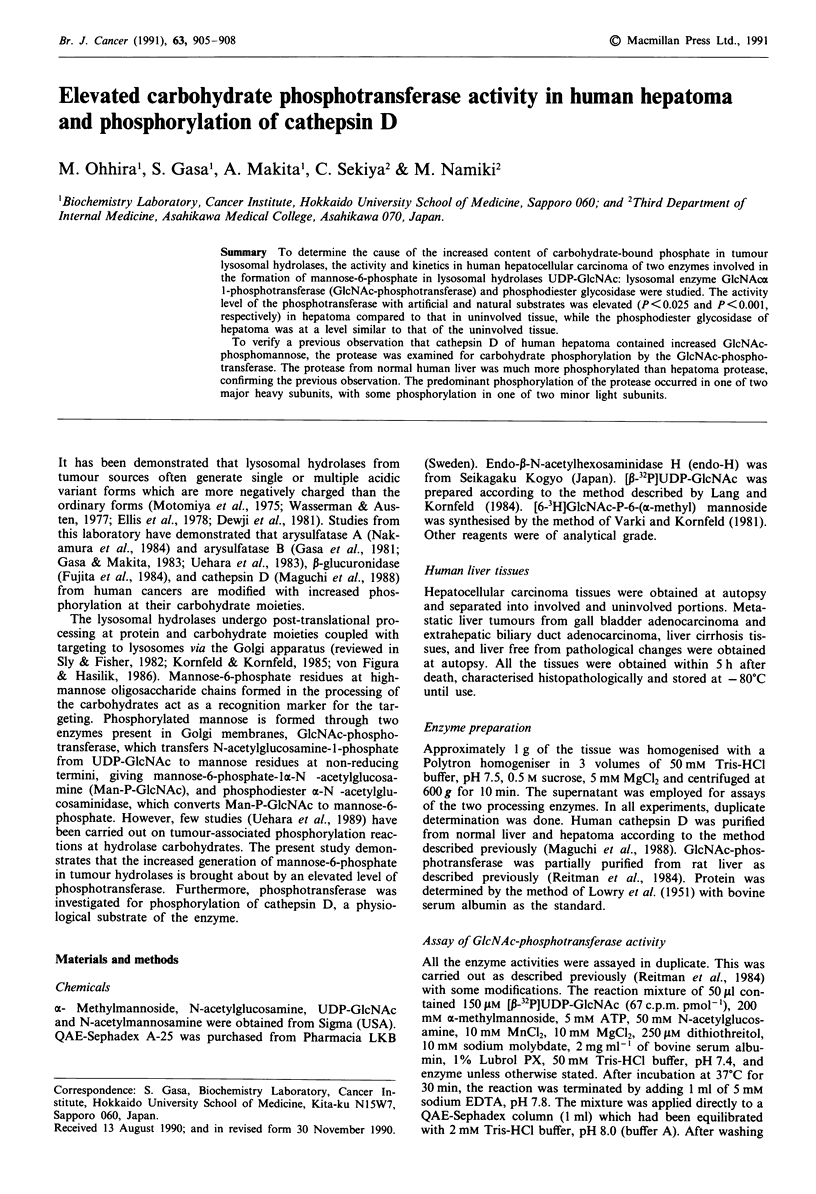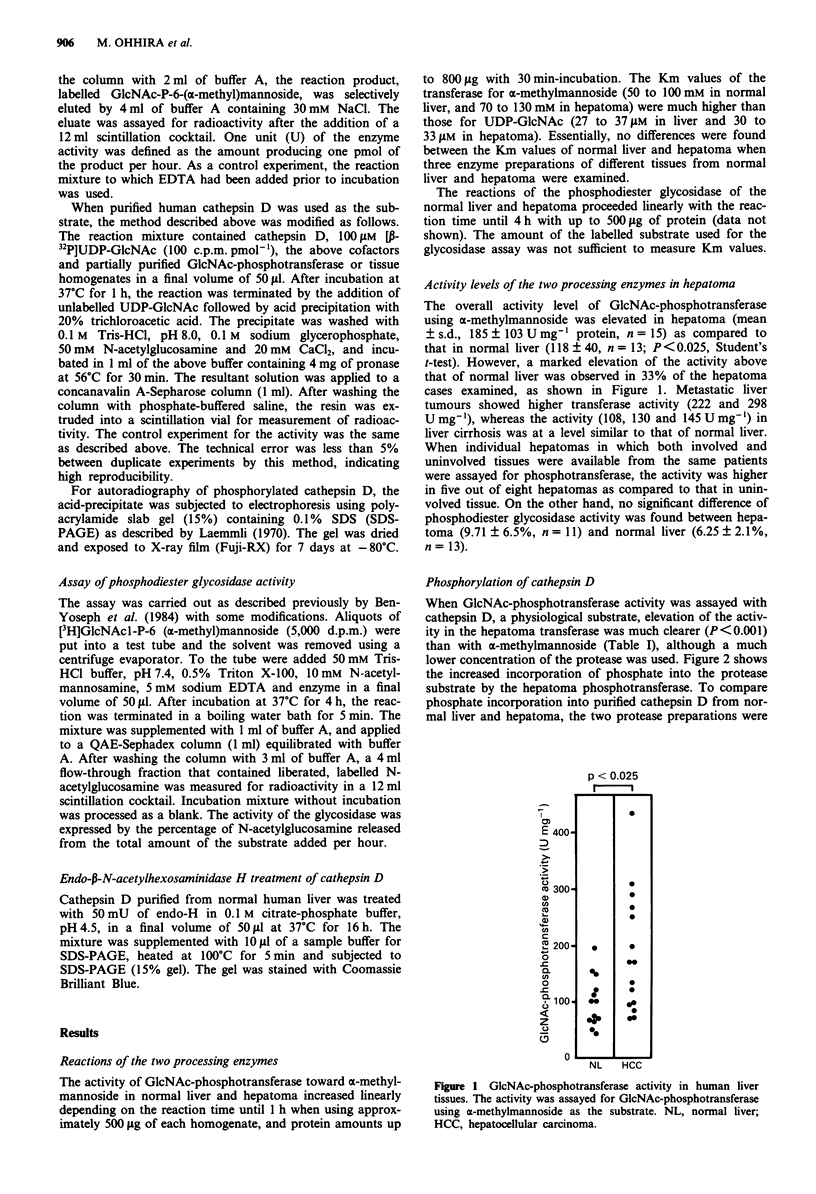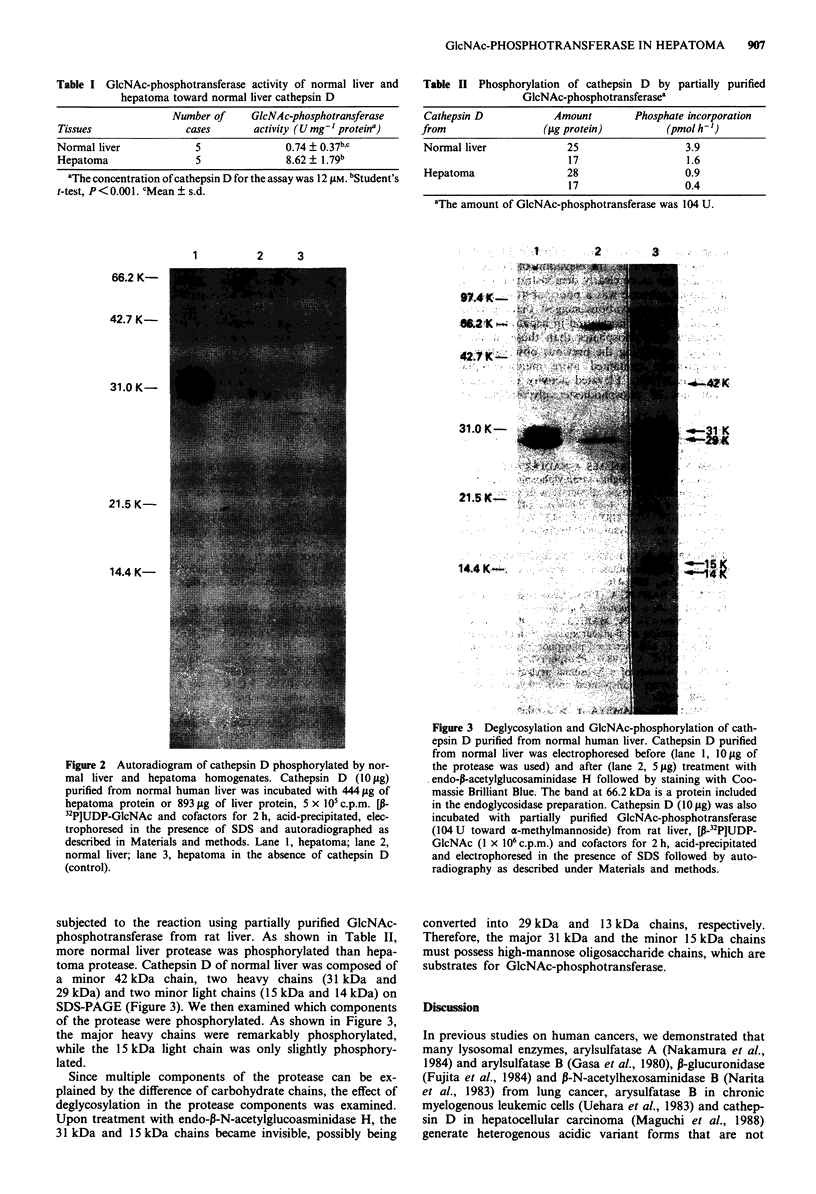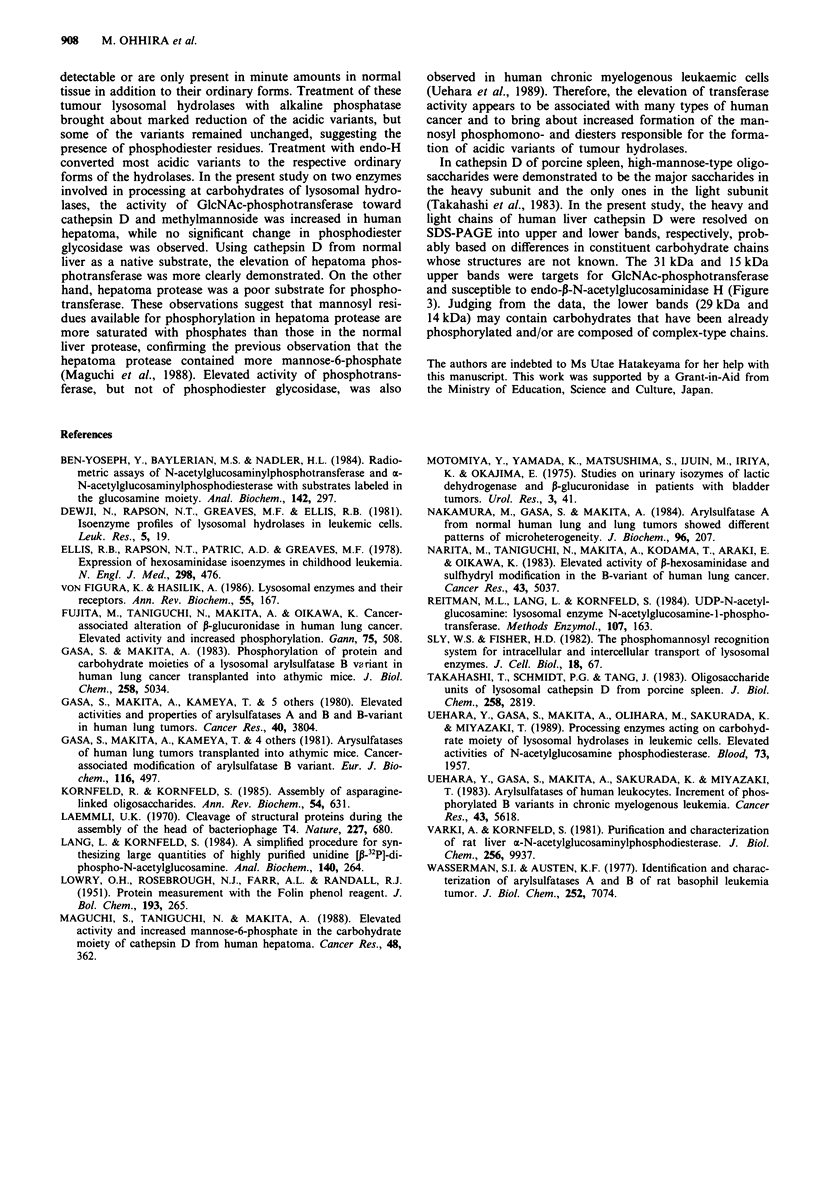# Elevated carbohydrate phosphotransferase activity in human hepatoma and phosphorylation of cathepsin D.

**DOI:** 10.1038/bjc.1991.199

**Published:** 1991-06

**Authors:** M. Ohhira, S. Gasa, A. Makita, C. Sekiya, M. Namiki

**Affiliations:** Biochemistry Laboratory, Hokkaido University School of Medicine, Sapporo, Japan.

## Abstract

**Images:**


Br. J. Cancer (1991), 63, 905-908 © Macmillan Press Ltd., 1991

### Elevated carbohydrate phosphotransferase activity in human hepatoma and phosphorylation of cathepsin D

M. Ohhira[1], S. Gasa[1], A. Makita[1], C. Sekiya[2] & M. Namiki[2]

[1]*Biochemistry Laboratory, Cancer Institute, Hokkaido University School of Medicine, Sapporo 060; and* [2]*Third Department of Internal Medicine, Asahikawa Medical College, Asahikawa 070, Japan.*

**Summary** To determine the cause of the increased content of carbohydrate-bound phosphate in tumour lysosomal hydrolases, the activity and kinetics in human hepatocellular carcinoma of two enzymes involved in the formation of mannose-6-phosphate in lysosomal hydrolases UDP-GlcNAc: lysosomal enzyme GlcNAcα 1-phosphotransferase (GlcNAc-phosphotransferase) and phosphodiester glycosidase were studied. The activity level of the phosphotransferase with artificial and natural substrates was elevated ($P<0.025$ and $P<0.001$, respectively) in hepatoma compared to that in uninvolved tissue, while the phosphodiester glycosidase of hepatoma was at a level similar to that of the uninvolved tissue.

To verify a previous observation that cathepsin D of human hepatoma contained increased GlcNAc-phosphomannose, the protease was examined for carbohydrate phosphorylation by the GlcNAc-phosphotransferase. The protease from normal human liver was much more phosphorylated than hepatoma protease, confirming the previous observation. The predominant phosphorylation of the protease occurred in one of two major heavy subunits, with some phosphorylation in one of two minor light subunits.

It has been demonstrated that lysosomal hydrolases from tumour sources often generate single or multiple acidic variant forms which are more negatively charged than the ordinary forms (Motomiya *et al.*, 1975; Wasserman & Austen, 1977; Ellis *et al.*, 1978; Dewji *et al.*, 1981). Studies from this laboratory have demonstrated that arysulfatase A (Nakamura *et al.*, 1984) and arysulfatase B (Gasa *et al.*, 1981; Gasa & Makita, 1983; Uehara *et al.*, 1983), β-glucuronidase (Fujita *et al.*, 1984), and cathepsin D (Maguchi *et al.*, 1988) from human cancers are modified with increased phosphorylation at their carbohydrate moieties.

The lysosomal hydrolases undergo post-translational processing at protein and carbohydrate moieties coupled with targeting to lysosomes *via* the Golgi apparatus (reviewed in Sly & Fisher, 1982; Kornfeld & Kornfeld, 1985; von Figura & Hasilik, 1986). Mannose-6-phosphate residues at high-mannose oligosaccharide chains formed in the processing of the carbohydrates act as a recognition marker for the targeting. Phosphorylated mannose is formed through two enzymes present in Golgi membranes, GlcNAc-phosphotransferase, which transfers N-acetylglucosamine-1-phosphate from UDP-GlcNAc to mannose residues at non-reducing termini, giving mannose-6-phosphate-1α-N -acetylglucosamine (Man-P-GlcNAc), and phosphodiester α-N -acetylglucosaminidase, which converts Man-P-GlcNAc to mannose-6-phosphate. However, few studies (Uehara *et al.*, 1989) have been carried out on tumour-associated phosphorylation reactions at hydrolase carbohydrates. The present study demonstrates that the increased generation of mannose-6-phosphate in tumour hydrolases is brought about by an elevated level of phosphotransferase. Furthermore, phosphotransferase was investigated for phosphorylation of cathepsin D, a physiological substrate of the enzyme.

### Materials and methods

### *Chemicals*

α- Methylmannoside, N-acetylglucosamine, UDP-GlcNAc and N-acetylmannosamine were obtained from Sigma (USA). QAE-Sephadex A-25 was purchased from Pharmacia LKB (Sweden). Endo-β-N-acetylhexosaminidase H (endo-H) was from Seikagaku Kogyo (Japan). [β-$^{32}$P]UDP-GlcNAc was prepared according to the method described by Lang and Kornfeld (1984). [6-$^{3}$H]GlcNAc-P-6-(α-methyl) mannoside was synthesised by the method of Varki and Kornfeld (1981). Other reagents were of analytical grade.

### *Human liver tissues*

Hepatocellular carcinoma tissues were obtained at autopsy and separated into involved and uninvolved portions. Metastatic liver tumours from gall bladder adenocarcinoma and extrahepatic biliary duct adenocarcinoma, liver cirrhosis tissues, and liver free from pathological changes were obtained at autopsy. All the tissues were obtained within 5 h after death, characterised histopathologically and stored at −80°C until use.

### *Enzyme preparation*

Approximately 1 g of the tissue was homogenised with a Polytron homogeniser in 3 volumes of 50 mM Tris-HCl buffer, pH 7.5, 0.5 M sucrose, 5 mM $MgCl_2$ and centrifuged at 600 *g* for 10 min. The supernatant was employed for assays of the two processing enzymes. In all experiments, duplicate determination was done. Human cathepsin D was purified from normal liver and hepatoma according to the method described previously (Maguchi *et al.*, 1988). GlcNAc-phosphotransferase was partially purified from rat liver as described previously (Reitman *et al.*, 1984). Protein was determined by the method of Lowry *et al.* (1951) with bovine serum albumin as the standard.

### *Assay of GlcNAc-phosphotransferase activity*

All the enzyme activities were assayed in duplicate. This was carried out as described previously (Reitman *et al.*, 1984) with some modifications. The reaction mixture of 50 μl contained 150 μM [β-$^{32}$P]UDP-GlcNAc (67 c.p.m. $pmol^{-1}$), 200 mM α-methylmannoside, 5 mM ATP, 50 mM N-acetylglucosamine, 10 mM $MnCl_2$, 10 mM $MgCl_2$, 250 μM dithiothreitol, 10 mM sodium molybdate, 2 mg $ml^{-1}$ of bovine serum albumin, 1% Lubrol PX, 50 mM Tris-HCl buffer, pH 7.4, and enzyme unless otherwise stated. After incubation at 37°C for 30 min, the reaction was terminated by adding 1 ml of 5 mM sodium EDTA, pH 7.8. The mixture was applied directly to a QAE-Sephadex column (1 ml) which had been equilibrated with 2 mM Tris-HCl buffer, pH 8.0 (buffer A). After washing

Correspondence: S. Gasa, Biochemistry Laboratory, Cancer Institute, Hokkaido University School of Medicine, Kita-ku N15W7, Sapporo 060, Japan.
Received 13 August 1990; and in revised form 30 November 1990.

the column with 2 ml of buffer A, the reaction product, labelled GlcNAc-P-6-(α-methyl)mannoside, was selectively eluted by 4 ml of buffer A containing 30 mM NaCl. The eluate was assayed for radioactivity after the addition of a 12 ml scintillation cocktail. One unit (U) of the enzyme activity was defined as the amount producing one pmol of the product per hour. As a control experiment, the reaction mixture to which EDTA had been added prior to incubation was used.

When purified human cathepsin D was used as the substrate, the method described above was modified as follows. The reaction mixture contained cathepsin D, 100 μM [β-$^{32}$P]UDP-GlcNAc (100 c.p.m. pmol$^{-1}$), the above cofactors and partially purified GlcNAc-phosphotransferase or tissue homogenates in a final volume of 50 μl. After incubation at 37°C for 1 h, the reaction was terminated by the addition of unlabelled UDP-GlcNAc followed by acid precipitation with 20% trichloroacetic acid. The precipitate was washed with 0.1 M Tris-HCl, pH 8.0, 0.1 M sodium glycerophosphate, 50 mM N-acetylglucosamine and 20 mM $CaCl_2$, and incubated in 1 ml of the above buffer containing 4 mg of pronase at 56°C for 30 min. The resultant solution was applied to a concanavalin A-Sepharose column (1 ml). After washing the column with phosphate-buffered saline, the resin was extruded into a scintillation vial for measurement of radioactivity. The control experiment for the activity was the same as described above. The technical error was less than 5% between duplicate experiments by this method, indicating high reproducibility.

For autoradiography of phosphorylated cathepsin D, the acid-precipitate was subjected to electrophoresis using polyacrylamide slab gel (15%) containing 0.1% SDS (SDS-PAGE) as described by Laemmli (1970). The gel was dried and exposed to X-ray film (Fuji-RX) for 7 days at −80°C.

### *Assay of phosphodiester glycosidase activity*

The assay was carried out as described previously by Ben-Yoseph *et al.* (1984) with some modifications. Aliquots of [$^3$H]GlcNAc1-P-6 (α-methyl)mannoside (5,000 d.p.m.) were put into a test tube and the solvent was removed using a centrifuge evaporator. To the tube were added 50 mM Tris-HCl buffer, pH 7.4, 0.5% Triton X-100, 10 mM N-acetylmannosamine, 5 mM sodium EDTA and enzyme in a final volume of 50 μl. After incubation at 37°C for 4 h, the reaction was terminated in a boiling water bath for 5 min. The mixture was supplemented with 1 ml of buffer A, and applied to a QAE-Sephadex column (1 ml) equilibrated with buffer A. After washing the column with 3 ml of buffer A, a 4 ml flow-through fraction that contained liberated, labelled N-acetylglucosamine was measured for radioactivity in a 12 ml scintillation cocktail. Incubation mixture without incubation was processed as a blank. The activity of the glycosidase was expressed by the percentage of N-acetylglucosamine released from the total amount of the substrate added per hour.

### *Endo-β-N-acetylhexosaminidase H treatment of cathepsin D*

Cathepsin D purified from normal human liver was treated with 50 mU of endo-H in 0.1 M citrate-phosphate buffer, pH 4.5, in a final volume of 50 μl at 37°C for 16 h. The mixture was supplemented with 10 μl of a sample buffer for SDS-PAGE, heated at 100°C for 5 min and subjected to SDS-PAGE (15% gel). The gel was stained with Coomassie Brilliant Blue.

### Results

### *Reactions of the two processing enzymes*

The activity of GlcNAc-phosphotransferase toward α-methylmannoside in normal liver and hepatoma increased linearly depending on the reaction time until 1 h when using approximately 500 μg of each homogenate, and protein amounts up to 800 μg with 30 min-incubation. The Km values of the transferase for α-methylmannoside (50 to 100 mM in normal liver, and 70 to 130 mM in hepatoma) were much higher than those for UDP-GlcNAc (27 to 37 μM in liver and 30 to 33 μM in hepatoma). Essentially, no differences were found between the Km values of normal liver and hepatoma when three enzyme preparations of different tissues from normal liver and hepatoma were examined.

The reactions of the phosphodiester glycosidase of the normal liver and hepatoma proceeded linearly with the reaction time until 4 h with up to 500 μg of protein (data not shown). The amount of the labelled substrate used for the glycosidase assay was not sufficient to measure Km values.

### *Activity levels of the two processing enzymes in hepatoma*

The overall activity level of GlcNAc-phosphotransferase using α-methylmannoside was elevated in hepatoma (mean ± s.d., 185 ± 103 U mg$^{-1}$ protein, $n = 15$) as compared to that in normal liver (118 ± 40, $n = 13$; $P < 0.025$, Student's *t*-test). However, a marked elevation of the activity above that of normal liver was observed in 33% of the hepatoma cases examined, as shown in Figure 1. Metastatic liver tumours showed higher transferase activity (222 and 298 U mg$^{-1}$), whereas the activity (108, 130 and 145 U mg$^{-1}$) in liver cirrhosis was at a level similar to that of normal liver. When individual hepatomas in which both involved and uninvolved tissues were available from the same patients were assayed for phosphotransferase, the activity was higher in five out of eight hepatomas as compared to that in uninvolved tissue. On the other hand, no significant difference of phosphodiester glycosidase activity was found between hepatoma (9.71 ± 6.5%, $n = 11$) and normal liver (6.25 ± 2.1%, $n = 13$).

### *Phosphorylation of cathepsin D*

When GlcNAc-phosphotransferase activity was assayed with cathepsin D, a physiological substrate, elevation of the activity in the hepatoma transferase was much clearer ($P < 0.001$) than with α-methylmannoside (Table I), although a much lower concentration of the protease was used. Figure 2 shows the increased incorporation of phosphate into the protease substrate by the hepatoma phosphotransferase. To compare phosphate incorporation into purified cathepsin D from normal liver and hepatoma, the two protease preparations were

**Figure 1** GlcNAc-phosphotransferase activity in human liver tissues. The activity was assayed for GlcNAc-phosphotransferase using α-methylmannoside as the substrate. NL, normal liver; HCC, hepatocellular carcinoma.

**Table I** GlcNAc-phosphotransferase activity of normal liver and hepatoma toward normal liver cathepsin D

| *Tissues* | *Number of cases* | *GlcNAc-phosphotransferase activity (U mg$^{-1}$ protein$^{a}$)* |
|---|---|---|
| Normal liver | 5 | 0.74 ± 0.37[b,c] |
| Hepatoma | 5 | 8.62 ± 1.79[b] |

[a]The concentration of cathepsin D for the assay was 12 μM. [b]Student's *t*-test, $P < 0.001$. [c]Mean ± s.d.

**Table II** Phosphorylation of cathepsin D by partially purified GlcNAc-phosphotransferase[a]

| *Cathepsin D from* | *Amount (μg protein)* | *Phosphate incorporation (pmol h$^{-1}$)* |
|---|---|---|
| Normal liver | 25 | 3.9 |
| | 17 | 1.6 |
| Hepatoma | 28 | 0.9 |
| | 17 | 0.4 |

[a]The amount of GlcNAc-phosphotransferase was 104 U.

**Figure 2** Autoradiogram of cathepsin D phosphorylated by normal liver and hepatoma homogenates. Cathepsin D (10 μg) purified from normal human liver was incubated with 444 μg of hepatoma protein or 893 μg of liver protein, $5 \times 10^5$ c.p.m. [β-$^{32}$P]UDP-GlcNAc and cofactors for 2 h, acid-precipitated, electrophoresed in the presence of SDS and autoradiographed as described in Materials and methods. Lane 1, hepatoma; lane 2, normal liver; lane 3, hepatoma in the absence of cathepsin D (control).

**Figure 3** Deglycosylation and GlcNAc-phosphorylation of cathepsin D purified from normal human liver. Cathepsin D purified from normal liver was electrophoresed before (lane 1, 10 μg of the protease was used) and after (lane 2, 5 μg) treatment with endo-β-acetylglucosaminidase H followed by staining with Coomassie Brilliant Blue. The band at 66.2 kDa is a protein included in the endoglycosidase preparation. Cathepsin D (10 μg) was also incubated with partially purified GlcNAc-phosphotransferase (104 U toward α-methylmannoside) from rat liver, [β-$^{32}$P]UDP-GlcNAc ($1 \times 10^6$ c.p.m.) and cofactors for 2 h, acid-precipitated and electrophoresed in the presence of SDS followed by autoradiography as described under Materials and methods.

subjected to the reaction using partially purified GlcNAc-phosphotransferase from rat liver. As shown in Table II, more normal liver protease was phosphorylated than hepatoma protease. Cathepsin D of normal liver was composed of a minor 42 kDa chain, two heavy chains (31 kDa and 29 kDa) and two minor light chains (15 kDa and 14 kDa) on SDS-PAGE (Figure 3). We then examined which components of the protease were phosphorylated. As shown in Figure 3, the major heavy chains were remarkably phosphorylated, while the 15 kDa light chain was only slightly phosphorylated.

Since multiple components of the protease can be explained by the difference of carbohydrate chains, the effect of deglycosylation in the protease components was examined. Upon treatment with endo-β-N-acetylglucoasminidase H, the 31 kDa and 15 kDa chains became invisible, possibly being converted into 29 kDa and 13 kDa chains, respectively. Therefore, the major 31 kDa and the minor 15 kDa chains must possess high-mannose oligosaccharide chains, which are substrates for GlcNAc-phosphotransferase.

### Discussion

In previous studies on human cancers, we demonstrated that many lysosomal enzymes, arylsulfatase A (Nakamura *et al.*, 1984) and arylsulfatase B (Gasa *et al.*, 1980), β-glucuronidase (Fujita *et al.*, 1984) and β-N-acetylhexosaminidase B (Narita *et al.*, 1983) from lung cancer, arysulfatase B in chronic myelogenous leukemic cells (Uehara *et al.*, 1983) and cathepsin D in hepatocellular carcinoma (Maguchi *et al.*, 1988) generate heterogenous acidic variant forms that are not detectable or are only present in minute amounts in normal tissue in addition to their ordinary forms. Treatment of these tumour lysosomal hydrolases with alkaline phosphatase brought about marked reduction of the acidic variants, but some of the variants remained unchanged, suggesting the presence of phosphodiester residues. Treatment with endo-H converted most acidic variants to the respective ordinary forms of the hydrolases. In the present study on two enzymes involved in processing at carbohydrates of lysosomal hydrolases, the activity of GlcNAc-phosphotransferase toward cathepsin D and methylmannoside was increased in human hepatoma, while no significant change in phosphodiester glycosidase was observed. Using cathepsin D from normal liver as a native substrate, the elevation of hepatoma phosphotransferase was more clearly demonstrated. On the other hand, hepatoma protease was a poor substrate for phosphotransferase. These observations suggest that mannosyl residues available for phosphorylation in hepatoma protease are more saturated with phosphates than those in the normal liver protease, confirming the previous observation that the hepatoma protease contained more mannose-6-phosphate (Maguchi et al., 1988). Elevated activity of phosphotransferase, but not of phosphodiester glycosidase, was also observed in human chronic myelogenous leukaemic cells (Uehara et al., 1989). Therefore, the elevation of transferase activity appears to be associated with many types of human cancer and to bring about increased formation of the mannosyl phosphomono- and diesters responsible for the formation of acidic variants of tumour hydrolases.

In cathepsin D of porcine spleen, high-mannose-type oligosaccharides were demonstrated to be the major saccharides in the heavy subunit and the only ones in the light subunit (Takahashi et al., 1983). In the present study, the heavy and light chains of human liver cathepsin D were resolved on SDS-PAGE into upper and lower bands, respectively, probably based on differences in constituent carbohydrate chains whose structures are not known. The 31 kDa and 15 kDa upper bands were targets for GlcNAc-phosphotransferase and susceptible to endo-β-N-acetylglucosaminidase H (Figure 3). Judging from the data, the lower bands (29 kDa and 14 kDa) may contain carbohydrates that have been already phosphorylated and/or are composed of complex-type chains.

The authors are indebted to Ms Utae Hatakeyama for her help with this manuscript. This work was supported by a Grant-in-Aid from the Ministry of Education, Science and Culture, Japan.

### References

BEN-YOSEPH, Y., BAYLERIAN, M.S. & NADLER, H.L. (1984). Radiometric assays of N-acetylglucosaminylphosphotransferase and α-N-acetylglucosaminylphosphodiesterase with substrates labeled in the glucosamine moiety. *Anal. Biochem.*, **142**, 297.

DEWJI, N., RAPSON, N.T., GREAVES, M.F. & ELLIS, R.B. (1981). Isoenzyme profiles of lysosomal hydrolases in leukemic cells. *Leuk. Res.*, **5**, 19.

ELLIS, R.B., RAPSON, N.T., PATRIC, A.D. & GREAVES, M.F. (1978). Expression of hexosaminidase isoenzymes in childhood leukemia. *N. Engl. J. Med.*, **298**, 476.

VON FIGURA, K. & HASILIK, A. (1986). Lysosomal enzymes and their receptors. *Ann. Rev. Biochem.*, **55**, 167.

FUJITA, M., TANIGUCHI, N., MAKITA, A. & OIKAWA, K. Cancer-associated alteration of β-glucuronidase in human lung cancer. Elevated activity and increased phosphorylation. *Gann*, **75**, 508.

GASA, S. & MAKITA, A. (1983). Phosphorylation of protein and carbohydrate moieties of a lysosomal arylsulfatase B variant in human lung cancer transplanted into athymic mice. *J. Biol. Chem.*, **258**, 5034.

GASA, S., MAKITA, A., KAMEYA, T. & 5 others (1980). Elevated activities and properties of arylsulfatases A and B and B-variant in human lung tumors. *Cancer Res.*, **40**, 3804.

GASA, S., MAKITA, A., KAMEYA, T. & 4 others (1981). Arysulfatases of human lung tumors transplanted into athymic mice. Cancer-associated modification of arylsulfatase B variant. *Eur. J. Biochem.*, **116**, 497.

KORNFELD, R. & KORNFELD, S. (1985). Assembly of asparagine-linked oligosaccharides. *Ann. Rev. Biochem.*, **54**, 631.

LAEMMLI, U.K. (1970). Cleavage of structural proteins during the assembly of the head of bacteriophage T4. *Nature*, **227**, 680.

LANG, L. & KORNFELD, S. (1984). A simplified procedure for synthesizing large quantities of highly purified unidine [β-$^{32}$P]-diphospho-N-acetylglucosamine. *Anal. Biochem.*, **140**, 264.

LOWRY, O.H., ROSEBROUGH, N.J., FARR, A.L. & RANDALL, R.J. (1951). Protein measurement with the Folin phenol reagent. *J. Bol. Chem.*, **193**, 265.

MAGUCHI, S., TANIGUCHI, N. & MAKITA, A. (1988). Elevated activity and increased mannose-6-phosphate in the carbohydrate moiety of cathepsin D from human hepatoma. *Cancer Res.*, **48**, 362.

MOTOMIYA, Y., YAMADA, K., MATSUSHIMA, S., IJUIN, M., IRIYA, K. & OKAJIMA, E. (1975). Studies on urinary isozymes of lactic dehydrogenase and β-glucuronidase in patients with bladder tumors. *Urol. Res.*, **3**, 41.

NAKAMURA, M., GASA, S. & MAKITA, A. (1984). Arylsulfatase A from normal human lung and lung tumors showed different patterns of microheterogeneity. *J. Biochem.*, **96**, 207.

NARITA, M., TANIGUCHI, N., MAKITA, A., KODAMA, T., ARAKI, E. & OIKAWA, K. (1983). Elevated activity of β-hexosaminidase and sulfhydryl modification in the B-variant of human lung cancer. *Cancer Res.*, **43**, 5037.

REITMAN, M.L., LANG, L. & KORNFELD, S. (1984). UDP-N-acetylglucosamine: lysosomal enzyme N-acetylglucosamine-1-phosphotransferase. *Methods Enzymol.*, **107**, 163.

SLY, W.S. & FISHER, H.D. (1982). The phosphomannosyl recognition system for intracellular and intercellular transport of lysosomal enzymes. *J. Cell. Biol.*, **18**, 67.

TAKAHASHI, T., SCHMIDT, P.G. & TANG, J. (1983). Oligosaccharide units of lysosomal cathepsin D from porcine spleen. *J. Biol. Chem.*, **258**, 2819.

UEHARA, Y., GASA, S., MAKITA, A., OLIHARA, M., SAKURADA, K. & MIYAZAKI, T. (1989). Processing enzymes acting on carbohydrate moiety of lysosomal hydrolases in leukemic cells. Elevated activities of N-acetylglucosamine phosphodiesterase. *Blood*, **73**, 1957.

UEHARA, Y., GASA, S., MAKITA, A., SAKURADA, K. & MIYAZAKI, T. (1983). Arylsulfatases of human leukocytes. Increment of phosphorylated B variants in chronic myelogenous leukemia. *Cancer Res.*, **43**, 5618.

VARKI, A. & KORNFELD, S. (1981). Purification and characterization of rat liver α-N-acetylglucosaminylphosphodiesterase. *J. Biol. Chem.*, **256**, 9937.

WASSERMAN, S.I. & AUSTEN, K.F. (1977). Identification and characterization of arylsulfatases A and B of rat basophil leukemia tumor. *J. Biol. Chem.*, **252**, 7074.